# Combination of Host-Associated *Rummeliibacillus* sp. and *Microbacterium* sp. Positively Modulated the Growth, Feed Utilization, and Intestinal Microbial Population of Olive Flounder (*Paralichthys olivaceus*)

**DOI:** 10.3390/biology12111443

**Published:** 2023-11-17

**Authors:** Su-Jeong Lee, So Hee Kim, Da-In Noh, Young-Sun Lee, Tae-Rim Kim, Md Tawheed Hasan, Eun-Woo Lee, Won Je Jang

**Affiliations:** 1 Biopharmaceutical Engineering Major, Dong-Eui University, Busan 47340, Republic of Korea; 2 Southeast Sea Fisheries Research Institute, National Institute of Fisheries Science, Tongyeong 53085, Republic of Korea; 3 Department of Aquaculture, Sylhet Agricultural University, Sylhet 3100, Bangladesh; 4 Core-Facility Center for Tissue Regeneration, Dong-Eui University, Busan 47340, Republic of Korea

**Keywords:** *Rummeliibacillus* sp., *Microbacterium* sp., olive flounder, probiotics, microbiota

## Abstract

**Simple Summary:**

Global concerns have been raised about the negative effects of antibiotic usage on aquaculture. Synthetic antibiotics have biological alternatives, namely, probiotics, which have the potential to improve fish growth, immunity, intestinal microbial population, and disease resistance. Host-associated probiotics are more adaptable and functional in the known intestinal environment, and two novel strains of *Rummeliibacillus* sp. and *Microbacterium* sp. were identified from the flounder (*Paralichthys olivaceus*) intestine. It is scientifically established that the effect of multiprobiotics was better than that of individual probiotics; as such, these two strains were administered in 1 × 10^8^ CFU/g feed at a 50:50 ratio. At the end of the feeding experiment, the growth and feed utilization of the probiotic group (Pro) were higher than those of the control fish. Although serum biochemical parameters did not change, the immune parameter myeloperoxidase increased in the combined probiotic-fed group. These two strains also increased the beneficial microbial abundance in the intestine of the Pro group compared with that in the control fish. Therefore, the identification of the two novel probiotic strains from the flounder intestine has provided a basis for conducting further research, and their combination can be supplemented in commercial flounder cultures in future applications.

**Abstract:**

Two novel strains of *Rummeliibacillus* sp. and *Microbacterium* sp. were identified from the intestine of olive flounder (*Paralichthys olivaceus*) and characterized in vitro as potential probiotics. Feeds without probiotic and with a 50:50 mixture of these two strains (1 × 10^8^ CFU/g feed) were denoted as the control and Pro diets, respectively. Three randomly selected tanks (20 flounders/tank, ~11.4 g each) were used for each diet replication. After 8 weeks of feeding, the growth and feed utilization of the flounder in the Pro group improved (*p* < 0.05) compared to the control. Among four immune parameters, only myeloperoxidase activity was elevated in the Pro group. Serum biochemistry, intestinal microbial richness (Chao1), and diversity (Shannon index) remained unchanged (*p* ≥ 0.05), but phylogenetic diversity was enriched in the Pro fish intestine. Significantly lower Firmicutes and higher Proteobacteria were found in the Pro diet; the genus abundance in the control and Pro was as follows: *Staphylococcus* > *Lactobacillus* > *Corynebacterium* and *Lactobacillus* > *Staphylococcus* > *Corynebacterium*, respectively. Microbial linear discriminant scores and a cladogram analysis showed significant modulation. Therefore, the combination of two host-associated probiotics improved the growth and intestinal microbial population of flounder and could be supplemented in the Korean flounder industry.

## 1. Introduction

The olive flounder (*Paralichthys olivaceus*) aquaculture industry has intensified in the Republic of Korea, and it needs a considerable amount of feed supply, high stocking density, and frequent water exchange. According to the KOSIS [1], in 2021, the production of this fish was 41,776 metric tons, which was 46.7% of the total marine fish production of 89,423 metric tons. Intensive aquaculture has also resulted in environmental degradation, and changes in the physical, chemical, and biological parameters of culture environment have made this fish susceptible to infectious pathogens and other opportunistic bacteria that cause outbreaks of infectious diseases. Synthetic antibiotics and chemical chemotherapeutics are used to control these diseases, but this approach results in the accumulation of antibiotic residues in the fish body; consequently, these residues can be transmitted to humans, produce antibiotic-resistant pathogens, and cause a mass killing of harmful and beneficial bacteria [2,3]. Prevention is better than cure; aquaculture scientists worldwide have expressed their concern about the indiscriminate antibiotic usage and advised the application of probiotics as an alternative to antibiotics to increase immunity and thus prevent infectious diseases.

Probiotics are live microorganisms that confer a health benefit on the host when they are administered in adequate amounts [4]. They secrete antagonistic compounds to kill pathogens, exclude infectious bacteria by competing for space in the intestine, improve digestive enzyme activities, increase innate immune parameters, serve as a source of different nutrients, and improve disease protection [5]. Some intestinal commensal bacteria produce biodegradable antibiotics, namely, nisin and bacteriocin, to eliminate external pathogenic bacterial threats; they are also strong candidates for host-associated probiotic identification and characterization. Moreover, in light of aquaculture ecological parameters, water temperature, and salinity, as well as to ensure higher viability and functionality in a harsh intestinal environment, host-associated probiotics are more suitable than commercial probiotics. Probiotics modulate the intestinal microbial population and decrease the load of pathogenic bacteria; a well-balanced intestinal microbial population helps maintain the immune physiology and well-being of aquaculture species [6,7].

Two novel bacterial strains, *Rummeliibacillus* sp. and *Microbacterium* sp., were isolated and identified from healthy flounder intestine. These two potential host-associated probiotic bacteria successfully passed different in vitro characterization tests, namely, antibiotic susceptibility, hemolysis activity, HT-29 cell line cytotoxicity, gastric juice and bile acid viability, antagonistic nature, different enzyme secretion, and prebiotic utilization (data not published). In flounder research, apart from flounder host-associated probiotics, Hasan et al. [8] provided a dietary supplement with *Bacillus* SJ-10 from jeotgal, Cha et al. [9] used commercial *B. subtilis* or *B. licheniformis* for feeding, and Kim et al. [10] administered *Lactococcus lactis* BFE920 from sprouts to increase production, innate immunity, and protection against infectious diseases. Few studies have focused on flounder host-associated probiotic supplementation and its effect on this fish species [11,12]. The metabolites of one probiotic influence the growth, survival, and functionality of others; therefore, multiprobiotics instead of individual probiotics are increasingly used day by day in aquaculture ventures. However, studies have yet to quantify the combined effects of two flounder intestinal bacteria, *Rummeliibacillus* sp. and *Microbacterium* sp., after they are used as a dietary supplement for this species culture.

Therefore, the objective of this research was fixed as the dietary application of a 50:50 combination of these two potential flounder intestinal probiotics (*Rummeliibacillus* sp. and *Microbacterium* sp.) to quantify their combined effects on *P. olivaceus* growth, feed utilization, and immune responses. Moreover, any changes in the serum biochemical parameters and intestinal microbial population were also estimated.

## 2. Materials and Methods

### 2.1. Fish Diet Preparation

Fish diet composition, preparation, and preservation were performed in accordance with the methods of Hasan et al. [13]. Briefly, feed ingredients were weighed; then, fish oil and distilled water (300 mL/kg) were added and mixed thoroughly. Potential intestinal probiotic strains of *Rummeliibacillus* sp. and *Microbacterium* sp. were suspended in 300 mL of distilled water (1.67 × 10^8^ CFU/mL/strain) to obtain stable 1 × 10^8^ CFU of total 2 probiotics per gram of feed and formulate a probiotic-inoculated “Pro” diet. Diet without any probiotic inoculation was known as control feed. The prepared diets were subsequently air-dried at room temperature and stored in sealed plastic bags at 4 °C.

### 2.2. Fish Collection, Husbandry, and Feeding Trial

Juvenile *P. olivaceus* were obtained from a private hatchery; before the feeding trial was initiated, all the fish were fed with the control diet for 2 weeks to acclimatize to the experimental environment. At the beginning, ~11.4 g/flounder was stocked into six tanks; each tank contained 20 fish and had a volume of 60 L. Three tanks were randomly assigned as replicates for each of the control and Pro diets. Fish were fed to apparent satiation twice a day (10:00 and 16:00) for 8 weeks. Water quality was regularly monitored, and stable environmental parameters were maintained as temperature, 20.0 ± 0.5 °C; salinity, 31 ± 1 ppt; dissolved oxygen, 6.0 ± 0.5 mg/L; pH, 7.3 ± 0.3; and water flow, 1.2 L/min.

### 2.3. Sample Collection and Analysis

At the end of 8 weeks, the fish were starved for 24 h, and all surviving fish in the tanks were captured and weighed. Afterward, nine fish per diet group were anesthetized with 500 μL/L of 2-phenoxyethanol (Sigma-Aldrich, St. Louis, MO, USA), and blood was collected using nonheparinized and heparinized syringes for serum preparation (clotted blood centrifugation at 16,000× *g* for 10 min) and respiratory burst (RB) analysis, respectively.

### 2.4. Quantification of Growth, Feed Utilization, Innate Immunity, and Serum Biochemical Parameters

After the feeding experiment was completed, growth parameters (final body weight [FBW], weight gain [WG%], and specific growth rate [SGR%]) and feed utilization parameters (feed conversion ratio [FCR] and protein efficiency ratio [PER]) were calculated using the recognized scientific equations.

For innate immune parameters, superoxide dismutase (SOD) activity in the serum was quantified using a SOD kit (K335-100, BioVision, Milpitas, CA, USA) in accordance with the manufacturer’s instructions. Briefly, 20 μL serum was used to inhibit the superoxide anion produced by xanthine and xanthine oxidase on a water-soluble tetrazolium dye. The reaction endpoint was read at 450 nm after 20 min reaction at 37 °C, and percentage inhibitions were calculated following the equations mentioned in the kit.

Serum myeloperoxidase (MPO) activity was estimated as described by Quade and Roth [14] with minor modifications. Initially, in a 96-well plate, 20 μL serum was diluted in 80 μL of Hanks’ balanced solution and then 35 μL of 20 mM 3,3′,5,5′-tetramethylbenzidine hydrochloride (Sigma-Aldrich, St. Louis, MO, USA) and 35 μL of 5 mM H_2_O_2_ were added to each well. After 2 min of incubation, 35 μL of 4 M H_2_SO_4_ was added to stop the color change and the absorbance read at 450 nm.

The generation of oxidative radicals by neutrophils involved in phagocytic activity during RB in flounder blood was measured using the nitroblue tetrazolium assay, as described by Anderson and Siwicki [15]. Serum antiprotease (AP) levels were estimated using the method outlined by Heo et al. [11].

Total glucose (TG), total cholesterol (TC), alanine aminotransferase (ALT), and aspartate aminotransferase (AST) were quantified using specific kits in BS-390 chemistry analyzer (Mindray Bio-Medical Electronics, Shenzhen, China) at the Core-Facility Center for Tissue Regeneration, Dong-Eui University (Busan, Republic of Korea).

### 2.5. Intestinal Microbiome Analysis

In accordance with the described method of Jang et al. [16], flounder intestinal microbiome analysis was performed using the whole intestine of nine fish per group. Briefly, total DNA was extracted using a PowerSoil DNA isolation kit (MoBio, Carlsbad, CA, USA), and its quality and quantity were measured with a NanoDrop spectrophotometer. MiSeq sequencing (Illumina, San Diego, CA, USA) and library construction were performed by the company TheMOAGEN Co., Ltd. (Daejeon, Republic of Korea). Primers were designed to target the V3 to V4 regions of the 16S rRNA sequence. Raw sequences were filtered for quality, denoised, and merged; chimeras were removed using the q2-dada2 plugin [17]. The differential abundance of microbial taxa in the intestine because of dietary difference was estimated via linear discriminant analysis effect size (LEfSe) analysis. A cladogram was prepared to illustrate the abundant taxa between Pro and control group fish.

### 2.6. Statistical Analysis

The normality and homogeneity of variance of the dataset were assessed using Shapiro–Wilk and Levene tests, respectively. Data were then subjected to one-way ANOVA using the IBM SPSS software (SPSS Inc., version 17.0, Chicago, IL, USA) following Student’s *t*-test. Results with *p* < 0.05 were considered statistically significant, and means are presented as mean ± standard deviation.

## 3. Results

### 3.1. Effects of the Combined Probiotic Strains on Growth, Feed Utilization, Innate Immunity, and Serum Biochemical Parameters

After 8 weeks of feeding, the diets inoculated with novel *Rummeliibacillus* sp. and *Microbacterium* sp. strains (Pro diet) positively influenced the growth parameters of olive flounder by significantly improving FBW, WG, and SGR compared with those of the control (Table 1).

Similarly, feed utilization parameters, such as FCR and PER, were modulated (*p* < 0.05) in the Pro group compared with those in the nonprobiotic diet-fed group.

No changes (*p* ≥ 0.05) were detected in serum biochemical parameters, such as ALT, AST, TG, and TC, between the Pro and control groups. Similarly, innate immune parameters, such as SOD, RB, and AP, remained unchanged after 8 weeks of feeding with the Pro diet. However, the MPO significantly increased in the Pro group compared with that in the control group (Table 2).

### 3.2. Effects of Combined Probiotics on the Alpha and Beta Diversity of the Intestinal Microbiota

**Figure 1:**
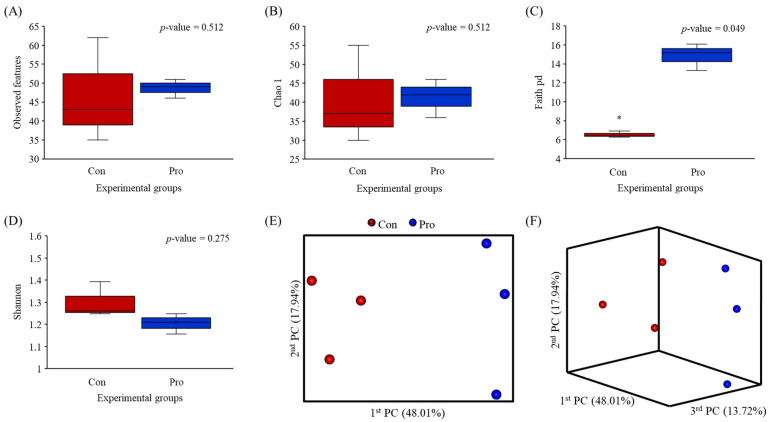
Alpha (**A**–**D**) and beta (**E**,**F**) diversity of the intestinal microbiota of olive flounder fed with the control and probiotic-inoculated diets for 8 weeks. Beta diversity as a principal coordinate analysis (PCoA) was performed on the basis of unweighted unifrac metrics. * *p* < 0.05.

After 8 weeks of feeding with the Pro diet, the observed species richness (Chao1 index) and diversity (Shannon index) remained unchanged (*p* ≥ 0.05) relative to the control. However, phylogenetic diversity (PD) was significantly higher in the combined host-associated potential probiotic-supplemented fish intestine than in the control intestine (Figure 1C).

Principal coordinate analysis (PCoA) based on unweighted unifrac metrics was used to analyze beta diversity (Figure 1E,F). The Pro and control intestinal microbiota differed, but the microorganisms in each group were closely associated.

### 3.3. Modulation of the Relative Abundance of Microbiota at Phylum and Genus Levels

**Figure 2:**
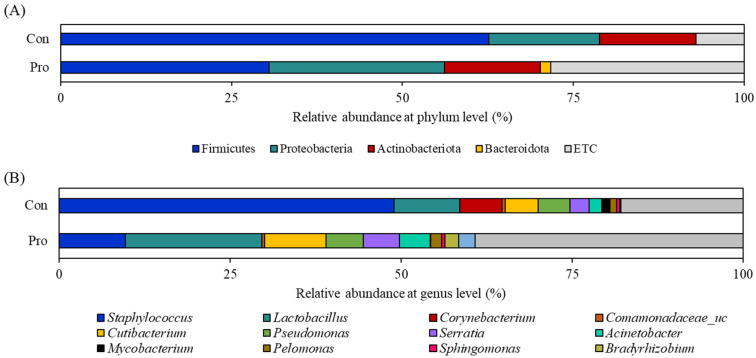
Relative abundance of the intestinal bacterial communities of olive flounder at the phylum (**A**) and genus (**B**) levels after supplementation with the control and probiotic-inoculated diets for 8 weeks.

At the phylum level, Firmicutes was the most abundant in the intestine of the control flounder, followed by Proteobacteria and Actinobacteria (Figure 2A). However, the abundance of Firmicutes was significantly lower and the abundance of Proteobacteria was higher in the intestine of the Pro group than in the intestine of the control group.

The relative abundance of the genus in the control group exhibited the following order: *Staphylococcus* > *Lactobacillus* > *Corynebacterium*; in the Pro group, the order was *Lactobacillus* > *Staphylococcus* > *Corynebacterium*. The abundance of *Lactobacillus* was significantly higher, and the abundance of *Staphylococcus* was significantly lower in the intestine of the fish fed with combined probiotics than in the intestine of the control fish (Figure 2B).

**Figure 3:**
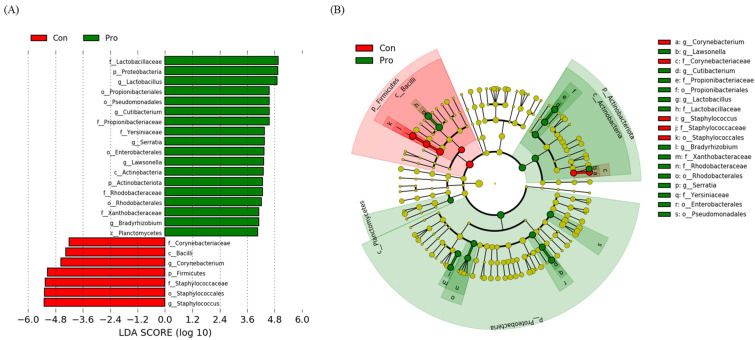
Linear discriminant analysis effect size (LEfSe) analysis of the differential abundance of taxa within the olive flounder intestinal microbiota following random sampling from each group. (**A**) Linear discriminant analysis (LDA) score of the abundance of taxa; (**B**) cladogram showing differentially abundant taxa of phylum to genus between the two groups.

The LDA scores of *Lactobacillaceae*, Proteobacteria, and *Lactobacillus* were > 4.8 in the Pro group; by comparison, Firmicutes, *Staphylococcaceae*, Staphylococcales, and *Staphylococcus* scores were > −4.8, which indicated the difference in the abundance of microbiota at the taxon level (Figure 3A). The cladogram (Figure 3B) demonstrated significant variations; that is, Firmicutes and Bacilli were dominant in the intestine of the control group, whereas Actinobacteria, Actinobacteriota, Proteobacteria, and Planctomyetes were dominant in the Pro group.

## 4. Discussion

Because of low pH and presence of different digestive and metabolic enzymes, the fish intestinal environment is challenging for maintaining the viability and functionality of supplemented probiotics. Thus far, different probiotics are provided as dietary supplements in commercially cultured species, and they are efficient in modulating growth, immunity, and intestinal microbial population. The combination of two or more probiotics is supplemented in aquaculture to achieve a synergistic effect because microbial metabolites produced by one probiotic may influence the performance of the other; as a result, the effect of combined probiotics is stronger than that of individual probiotics [18]. Nayak [19] mentioned that the survival and activity of the sustained microbial community of the gastrointestinal tract of fish fed with multispecies probiotics are higher than those of fish fed with single-species probiotics.

In this research, equal concentrations of the Pro diet inoculated with host-associated *Rummeliibacillus* sp. and *Microbacterium* sp. improved the growth and feed utilization of olive flounder. These types of positive parameter modulations were previously detected after dietary administration with multiprobiotics composed of *Bacillus subtilis* and *B. licheniformis* in Nile tilapia (*Oreochromis niloticus*) [20]; *Bacillus* sp., *Pedicoccus* sp., *Enterococcus* sp., *Lactobacillus* sp., and *Pediococcus acidilactici* in rainbow trout (*Oncorhynchus mykiss*) [21]; and *Enterococcus faecium* QAUEF01 and *Geotrichum candidum* QAUGC01 in rohu fish (*Labeo rohita*) [22]. However, feeding a mixture of *B. licheniformis* SK3927, *B. amyloliquefaciens* SK4079, *B. subtilis* SK4082, *L. brevis* SK1751, *L. plantarum* SK3494, and *Saccharomyces cerevisiae* SK3587 has failed improve the growth and feed utilization of flounder [23]. Probiotics can produce different essential enzymes that can increase digestive enzyme activities in the intestine to accelerate diet digestion and acclimation, resulting in increased feed utilization and growth performance [24,25]. In this present study, the actual underlying mechanism of growth and feed utilization modulation by *Rummeliibacillus* sp. and *Microbacterium* sp. was unknown, and digestive enzymes were not quantified; however, both these novel strains produced six different enzymes in vitro (API test), which might increase the intestinal enzyme actions for the enhanced feed utilization, thereby improving growth performance.

ALT and AST are stress enzymes secreted from the liver to the blood stream when fish are exposed to toxicants and subjected to cellular degradation [26]. Under stress conditions, fish require energy that can be provided by glucose production; as a stress indicator, the presence of increased glucose levels in the blood indicates that fish is exposed to stress [27]. In this study, serum biochemical parameters, specifically ALT, AST, and TG, had no significant differences after the fish were fed with the combination of the two novel probiotic strains; therefore, these two potential probiotics had no toxic or harmful effects on the flounder culture. In fish, the innate immune system is composed of cellular components, humoral parameters, and epithelial or mucosal barrier [28]. Cytotoxic cells, macrophages, monocytes, natural killer cells, and neutrophils are considered phagocytic cells [29], and humoral innate immunity involves bacteriolytic or hemolytic enzymes [19] that nonspecifically kill or destroy invading pathogens. In this innate immune system, phagocytic cells become activated during RB, and consumed oxygen is converted to superoxide (O_2_^−^), and MPO transforms O_2_^−^ to hypochlorous acid (HClO) [30]. O_2_^−^ and HClO are detrimental to invading infectious pathogens and involved in their elimination from the fish body [31]. In the present study, MPO in the Pro diet-fed flounder was significantly higher than that in the control fish, indicating that the immunological status of the Pro-fed fish was better than that of the nonprobiotic-fed group. The dietary application of probiotics improves immunological status [8,23,32,33] and does not elicit harmful effects [13,34,35,36] on different commercially cultured species, including flounder, thus supporting the current findings.

Pérez-Sánchez et al. [5] indicated that the intestinal microbial community of fish plays a vital role in host nutrient digestion, absorption, and assimilation, as well as metabolism, physiological status, pathological defense, and innate immune system activation. The fish intestinal microbiome can be modulated by temperature, environment, diet composition and dietary supplements, and different developmental stages [37]. In the present study, the 50:50 combination of two potential probiotics was unable to change the intestinal microbial diversity, richness, and observation, but phylogenetic diversity in the Pro group was improved. Differences between the Pro and control diets were observed through using PCoA, and the microbiota of the individual feeding group were closely associated. Beta diversity normally estimates the community similarity or distance between/among samples; in the present study, closeness in the Pro diet group may be an indicator of microbial implantation through *Rummeliibacillus* sp. and *Microbacterium* sp. supplementation. Moreover, the significant decrease in Firmicutes and the significant increase in Proteobacteria at the phylum level of the Pro fish intestine validated the phylogenetic diversity estimated by using the alpha diversity analysis. Firmicutes and Proteobacteria are the most abundant phyla in cultured and wild fine flounder (*P. adspersus*), respectively [38]. Dietary application with *Bacillus* ap. SJ-10 probiotic increases the Proteobacteria concentration compared with that in the control *P. olivaceus* [39]. Beneficial *Lactobacillus* concentrations increased in Pro-fed fish by decreasing the concentration of harmful *Staphylococcus* compared with those in the nonprobiotic-supplemented fish. *Staphylococcus* may cause soft skin infections, such as abscesses, furuncles, and cellulites, and *S. aureus* causes infection in the blood [40]. In the present study, the abundance of microbial taxa (LDA score) and the presence of Actinobacteria and Proteobacteria (cladogram observation) were higher in the treatment. Actinobacteria are known to produce different secondary metabolites [41,42]. The increased proportions of natural antibiotics (nisin and bacteriocin) producing *Lactobacillus* in the intestine of probiotic-fed fish were consistent with these phylum-level findings. Additionally, it is not confirmed, but the combination of *Rummeliibacillus* sp. and *Microbacterium* sp. might make the intestinal environment favorable to colonizing different microbes, thereby increasing taxon abundance in Pro-fed fish intestine. The application of probiotics through diet positively improves the beneficial bacteria in the intestinal microbial community of commercially cultured fish species [32,43,44,45], and this observation is consistent with the present findings.

## 5. Conclusions

This research demonstrates that the combination of two flounder host-associated probiotics positively affected the different aspects of this species. The increase in feed utilization with these novel strains means less feed application for increased production, resulting in farmers’ profitability. Changes in some innate immune parameters are likely to improve disease prevention, ultimately eliminating antibiotics from the flounder culture. Moreover, these host-associated strains can increase the beneficial bacteria in the intestine by decreasing pathogenic concentrations. In the future, these two probiotics may be supplemented in the Korean aquaculture industry to resolve the current problems. However, a small-scale earthen pond experiment may be needed to observe variations in laboratory and field experiments before these probiotics can be applied in large aquaculture ventures.

## Figures and Tables

**Table 1:** Growth performance and feed utilization of olive flounder supplemented with experimental feed additives.

Groups	Growth Performance, Feed Utilization, and Organosomatic Parameters				
	FBW (g)	WG (%)	SGR (% day^−1^)	FCR	PER
Con	31.55 ± 0.50 ^a^	192.95 ± 6.79 ^a^	1.92 ± 0.04 ^a^	1.23 ± 0.04 ^b^	1.60 ± 0.08 ^a^
Pro	35.98 ± 1.05 ^b^	233.31 ± 6.80 ^b^	2.15 ± 0.04 ^b^	1.02 ± 0.04 ^a^	1.93 ± 0.11 ^b^

Values are mean ± SD of three replicates. Values with different superscript letters within the same column in the table are significantly different (*p* < 0.05). FBW, Final body weight = Final weight of total fish in tank/Fish number; WG, Weight gain = [(Final weight − Initial weight)/Initial weight] × 100; SGR, Specific growth rate = [(ln final weight − ln initial weight)/days] × 100; FCR, Feed conversion ratio = Dry feed intake/Wet body weight gain; PER, Protein efficiency ratio =Wet weight gain/Protein fed.

**Table 2:** Serum biochemical and nonspecific immune parameters of olive flounder supplemented with experimental feed additives.

**Groups**	**Serum Biochemical Parameters**			
	**ALT**	**AST**	**TG**	**TC**
Con	108.33 ± 14.57	46.33 ± 5.69	16.67 ± 2.08	147.33 ± 12.22
Pro	102.33 ± 16.26	45.33 ± 3.21	17.00 ± 3.61	138.67 ± 9.71
**Groups**	**Nonspecific immune parameters**			
	**SOD**	**RB**	**MPO**	**AP**
Con	37.11 ± 5.38	0.52 ± 0.03	0.46 ± 0.07 ^a^	72.33 ± 6.81
Pro	41.68 ± 4.83	0.68 ± 0.11	1.19 ± 0.22 ^b^	81.67 ± 9.07

Values are mean ± SD of three replicates. Values with different superscript letters within the same column in the table are significantly different (*p* < 0.05). No superscript letter indicates no significant difference (*p* > 0.05). ALT, Alanine aminotransferase (U/L); AST, Aspartate aminotransferase (U/L); TG, Total glucose (mg/dL); TC, Total cholesterol (mg/dL); SOD, Superoxide dismutase (% superoxide inhibition); RB, Respiratory burst (absorbance at 540 nm); MPO, Myeloperoxidase activity (absorbance at 450 nm); AP, Antiprotease activity (% of trypsin inhibition).

## Data Availability

The datasets used and/or analyzed during the current study are available from the corresponding author on reasonable request.
